# Democratizing water monitoring: Implementation of a community-based qPCR monitoring program for recreational water hazards

**DOI:** 10.1371/journal.pone.0229701

**Published:** 2020-05-13

**Authors:** Sydney P. Rudko, Ronald L. Reimink, Bradley Peter, Jay White, Patrick C. Hanington

**Affiliations:** 1 School of Public Health, University of Alberta, Edmonton, Alberta, Canada; 2 Office of Campus Ministries, Hope College, Holland, Michigan, United States of America; 3 Freshwater Solutions, LLC, Traverse City, Michigan, United States of America; 4 Alberta Lake Management Society, Edmonton, Alberta, Canada; 5 Aquality Environmental Consulting Ltd., Edmonton, Alberta, Canada; University of Helsinki, FINLAND

## Abstract

Recreational water monitoring can be challenging due to the highly variable nature of pathogens and indicator concentrations, the myriad of potential biological hazards to measure for, and numerous access points, both official and unofficial, that are used for recreation. The aim of this study was to develop, deploy, and assess the effectiveness of a quantitative polymerase chain reaction (qPCR) community-based monitoring (CBM) program for the assessment of bacterial and parasitic hazards in recreational water. This study developed methodologies for performing qPCR ‘in the field,’ then engaged with water management and monitoring groups and tested the method in a real-world implementation study to evaluate the accuracy of CBM using qPCR both quantitatively and qualitatively. This study found high reproducibility between qPCR results performed by non-expert field users and expert laboratory results, suggesting that qPCR as a methodology could be amenable to a CBM program.

## Introduction

Community-based monitoring is now routinely used for conservation and environmental monitoring [1]. Citizen science describes both a methodology of conducting large-scale research by recruiting volunteers and refers to the process by which citizens are involved in scientific investigation as researchers. Citizen science can include community-based monitoring (CBM) as a process of collaboration between government, industry, academia, and local community groups to monitor, track, and respond to issues [2–4].

The earliest incarnations of citizen science and CBM relied on volunteers as data collectors, but the discipline of CBM has grown and evolved. Recent arguments in favour of CBM suggest the field move away from a paradigm of “using citizens to do science” to an equal power relationship that views citizens as scientists, embracing some of the ideals of participatory action research [5]. CBM is poised to improve environmental decision-making. Its’ use has been on the rise due to budgetary constraints in both government and academia and because CBM can be a powerful methodology for generating large spatial or temporal datasets for monitoring/surveillance purposes. CBM improves scientific literacy, builds social capital, improves participation in local issues and benefits the environment [6,7]. Traditional CBM programs have typically relied on volunteers to conduct biodiversity surveys, conduct simple tests (i.e. Secchi disk tests for assessing water clarity), or specimen collection and shipment to central facilities for analysis. However, modern monitoring methods conducted in academia, industry, and government have evolved considerably to include large-scale spatial assessment methods, for example, algal/cyanobacteria bloom-tracking satellites, next-generation sequencing analysis, and eDNA monitoring. CBM programs also must evolve and advance as new technologies become available. In water monitoring, especially, quantitative polymerase chain reaction (qPCR) has emerged as a common method to conduct regulatory testing for sewage impacted recreational water (i.e. USEPA Method 1611), and a common screening method for fecal indicator organisms [8].

Quantitative PCR methods for the detection of surrogates and hazards in water have existed for decades and can be used to detect minute quantities of an organisms’ DNA in a complex matrix such as water, soil, or blood. qPCR is highly sensitive and is very specific for particular regions of DNA. In the last decade, agencies responsible for monitoring the environment and health have begun to capitalize on the potential of qPCR. Some of the greatest strides have been made in health, especially after the USEPA EMPACT study, which found that levels of enterococcus as measured by qPCR correlate with the risk of human gastrointestinal illness, and in correlating the amount of human-associated *Bacteroides* with human health targets [9,10,11]. Screening for toxigenic cyanobacteria species is also moving towards molecular detection methods. For example, in Poland, initial screening for toxin genes in recreational waters is conducted using qPCR, followed by immunochemical analysis to quantify the toxins [12]. In related fields like environmental monitoring, some locales have moved to molecular methods for monitoring for the veliger stage of invasive zebra (*Dreissena polymorpha*) and quagga (*Dreissena rostriformis bugensis*) mussels [13,14].

As the effectiveness of qPCR diagnostic tests continues to be realized, it is apparent qPCR is an excellent choice for CBM. qPCR is a platform; once the infrastructure is in place, monitoring for additional targets, or changing targets if new issues arise is as simple as validating a new test. For this reason, qPCR and related molecular techniques have been touted as grand solutions for point of care diagnostics in infectious disease monitoring, yet this future has not yet been realized [15,16]. The idea of portable diagnostic technologies that can be used to detect multiple targets feeding information into a surveillance system is attractive for a number of reasons; however, the development to implementation gap is often wider than one would expect.

It is often presumed that highly skilled personnel are required to execute molecular biology methods such as qPCR. Additionally, technologies to conduct testing portably have only just begun to emerge onto the market and have not been fully vetted. This study is, to our knowledge, the first of its kind to test the rigour of qPCR for detection/quantification of biological hazards and their surrogates in water through a CBM-implementation study. Here, we test the feasibility, reproducibility and reliability of implementing portable qPCR water monitoring amongst a variety of groups (government, NGO, and private enterprise). This was assessed both quantitatively, by conducting our own measurements on CBM partner samples, and qualitatively, through surveying our user groups to capture their perceptions of the technology and its fit within their individual contexts and organizations.

## Materials and methods

### Ethics statement

All procedures performed in studies involving human participants were in accordance with the ethical standards of the institutional and/or national research committee and with the 1964 Helsinki declaration and its later amendments or comparable ethical standards. This research was approved by the University of Alberta Human Research Ethics Board: Approval # Pro00048511. Oral consent was obtained from all survey participants.

### Implementation study design

**Fig 1:**
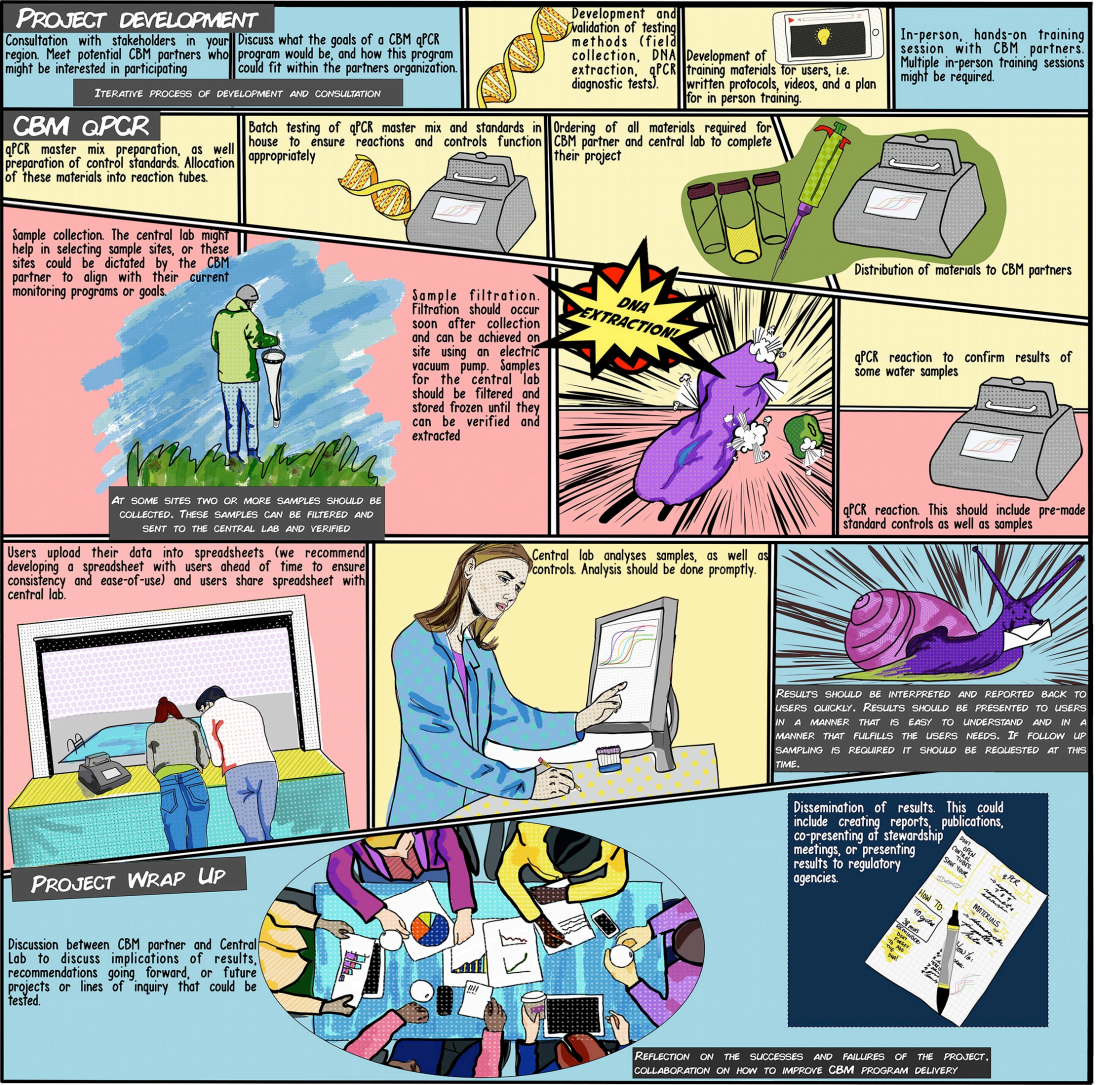
Implementation process of the CBM qPCR program. Cells with blue backgrounds are processes done in collaboration between the central laboratory and the CBM partners, yellow backgrounds indicate processes completed by the central laboratory, and red backgrounds indicate processes completed by the CBM partners.

We first connected with relevant stakeholders of recreational water in Alberta, and worked with them to determine their monitoring goals. Using a participatory research (PAR) approach, we then developed qPCR tests and testing methodologies that would fill these needs [17]. Under this PAR approach, CBM partners selected study sites they felt would be appropriate, and we advised and assisted in this selection where it seemed appropriate. In total, CBM partners conducted 985 qPCR tests over the two years of this program. Since the goal of this study was to measure the effectiveness of a CBM monitoring program in a real-world context, participants in the study were instructed to collect a duplicate sample or cut (using disposable, individually packaged sterile scalpel blades [Integra™ Miltex® No. 4]) the filter membrane in half after filtration and send this to the university laboratory. CBM partners stored their membranes frozen, and samples were transported to the laboratory on ice. Samples were picked up weekly or monthly, depending on sampling frequency. Partners in Michigan shipped samples overnight to Alberta. Samples in our laboratory would be processed in an identical fashion to the field user to compare novice versus expert methodologies (Fig 1) as soon as they were received; this was done to monitor method agreement. Additionally, CBM partners sent their extracted DNA to our laboratory, which enabled us to also perform qPCR on their DNA extracts and to perform inhibition reactions. CBM partners were encouraged to extract DNA from their water samples as soon as possible but stored filter membranes in the freezer if they weren’t going to perform extraction right away. Extracted DNA was also stored in the freezer until use, which was within one week of sample collection.

### Training of CBM partners

CBM partners were provided with a training video and a written protocol detailing all stages of the method, from sampling DNA extraction and qPCR. Training included using all equipment that was distributed, including thermocyclers and pipettes. Additionally, they were provided with two in-person training sessions. Typically, we would demonstrate the method in our laboratory, and the second training session would on-site at their location, where the CBM partner would run their first samples. As partners processed their samples, the laboratory would also perform DNA extractions and qPCR on the other half of the sample. These results were then compared. If the partner results disagreed with laboratory results, retraining occurred. CBM partners were also instructed on maintaining a clean workspace. This included the use of bleach to disinfect benchtops where DNA extraction occurred, using filter-tipped pipettes, and how to properly dispose of all materials.

### Sample collection

Specific water collection methods are detailed below for each target of interest; regardless of the volume collected, all samples were then filtered through a 0.4 μm polycarbonate filter (Pall FMFNL1050) using an electric vacuum pump (Vaccubrand ME1C). CBM partners had the option of either collecting and filtering a duplicate water sample for analysis, or cutting their filter membranes in half to be analyzed at the university laboratory.

#### Avian schistosome monitoring

Sample collection was conducted as described in Rudko et al. (2018). Briefly, 25L water samples, collected one litre at a time across a shoreline up to ~1m deep were passed through a 20μm plankton tow. Debris from inside was washed down using well water (this is not a contamination risk when monitoring for avian schistosomes as these parasites are shed from snail hosts, and only when those snail hosts also co-occur with locations where the bird definitive host’s feces are also present [18]) followed by a 95% ethanol wash and collection in sterile 50-mL conical tubes.

#### Toxin-producing cyanobacteria monitoring

Sample collection was conducted from watercraft operated by CBM partners on various lakes. Samples were collected through a one-way foot valve attached to weighted 3/4” Nalgene tubing. Samples were only collected from the euphotic zone as determined by a Secchi disk measurement at each lake’s deepest point. Ten sampling locations were selected for each lake, with water being composited from each sampling location into a central container. Water from this container was then poured into 50-mL conical tubes. Equipment was decontaminated between lakes using quaternary ammonium compound (QUAC) to prevent contamination between lakes (1500ppm QUAC for 10 minutes). QUAC disinfectants are membrane-active compounds that interact with the cytoplasmic membranes of eukaryotes and bacteria. Additionally, they bind DNA, making them useful disinfectants for samples that will be used for qPCR [19,20].

#### HF183 monitoring

All samples were collected by scooping two 50ml samples in sterile, conical, collection tubes from the surface water 15m from shore every 150m along the entire perimeter of each participating lake.

### DNA extraction

#### Onsite DNA extraction

DNA extraction was conducted using the MI Sample Prep Kit (Biomeme, USA) according to the manufacturers’ instructions. The MI sample prep kit is designed to function in the field. Lysis is accomplished by placing a filter in the lysis buffer and shaking for one minute. Next, the solution is passed through a syringe unit fitted with a DNA binding column. The column undergoes two washes to remove proteins and salts and then is dried using an acetone buffer before elution. Sample blanks were conducted by partners every batch of 24 samples processed. In 2018, the avian schistosomes monitoring group was interested in transitioning to a DNA extraction method that would allow for batch processing of samples. We, therefore, opted to transition their program to the DNeasy DNA extraction kit (Rudko et al. 2018). To set up this remote laboratory in a cost-effective manner, equipment (centrifuge, heating block, and vortex) were sourced from Dot Scientific (USA) (S2 Table), and pipettes were from the company VWR (USA).

### qPCR methods

#### Maintaining workflows

All master mix components were mixed in a cleanroom located at the University of Alberta and aliquoted into 0.2 ml thin-wall PCR tubes (Axygen, USA). All plasmid dilutions and preparation of positive controls occurred in a dead box, a PCR workspace designed to limit airflow and prevent cross-contamination between wells during reaction set up. Standards and reaction tubes were prepared independently to prevent cross-contamination. IDT DNA (USA) Prime Time Gene Expression Master mix was utilized for the field and in-laboratory qPCR because it is both light and temperature stable. That being said, all premixed reactions were stored frozen and transported on ice. Master mix was prepared and delivered to partners monthly.

#### In laboratory qPCR method

Samples were quantitated relative to a plasmid standard curve, which contained 50,000, 5000, 500, 50, 5 and 0.5 gene copies. Each of the gene targets below was synthesized (IDT DNA) into a puc19 plasmid vector (Genscript. USA). Thermocycling was performed on the ABI 7500 Fast or the QuantStudio 3 using a standard, 40 cycle, two-step reaction. The thermocycling parameters were a 30-second hold at 95**°**C, followed by a 30-second denaturation cycle at 95**°**C, and a 60**°**C annealing cycle. Each qPCR reaction had a final volume of 20μL, and we added 5μL of DNA to each reaction.

#### Avian schistosomes

The *18S* avian schistosomes-targeting qPCR assay was performed as described in Rudko et al. (2018) [21] (S1 Table). The LOD_95_ of this technique is 3.4 gene copies/ rxn [21]. qPCR master mix (IDT DNA) containing 1x master mix, and 200nm forward reverse primer and a fluorescein-labelled probe were used.

#### Toxigenic (*mcyE* gene) cyanobacteria monitoring

The *mcyE* gene targeting qPCR assay was performed as described in Qiu et al. (2013) [22] and Sipari et al. (2010) [23] (T S1 Table). The LOD_95_ of this technique is 6.25 copies/5μL. qPCR master mix (IDT DNA), containing 1x master mix, and 200nm forward reverse primer and 125nm fluorescein-labelled probe was used.

#### HF183 Bacteroides monitoring

This 16S gene-targeting assay was performed as described in Haugland et al. (2010). The LOD_95_ of this technique is 7.2 gene copies/rxn. qPCR master mix (IDT DNA), containing 1x master mix, and 100nm forward reverse primer and 80nm fluorescein-labelled probe was used (S1 Table).

#### In-field qPCR method

Mastermix components and concentrations were unchanged between the laboratory method and the field method, nor were the thermocycling parameters. CBM partners received four control tubes, which consisted of a negative control, and three standards (5000, 500, and 50 copies). They were instructed not to open these tubes to prevent contamination. CBM partners also received 12 tubes to add their own samples DNA to (Fig 1).

#### Inhibition controls

Inhibition controls were performed utilizing the inhibition control assay described in Rudko et al. (2017) [24]. Internal control plasmid DNA was spiked in excess into qPCR reactions containing 5μL of water sample DNA, and inhibition was defined as a 3-ct (i.e. 1 log) shift in amplification.

#### Creation of the field kits

Field kits given to CBM partners contained: the M1 DNA extraction kit (Biomeme), 1.5 ml snap-cap tubes, sample collection vials (Corning), a 20-micron plankton tow (Aquatic Research Instruments, USA), 0.45 μM polycarbonate filter funnels (Pall, FMFNL 1050), a 20 μL pipette, a box of pipette tips, PCR tubes, a laptop (Acer [Taiwan,] and Chromebooks [USA] were distributed) an Open qPCR (USA) thermocycler, all the necessary cables, and reaction strips (Fig 1, S3 Table).

### Capturing CBM partners perceptions of the method

CBM partners (six in total) were administered a survey with open-ended questions regarding the implementation of the method (S4 Table). All six CBM partners submitted a completed survey. Surveys were blinded from the researchers to encourage honesty from participants; a research associate received the surveys via email and edited them to remove any personal identifiers before sending them to the analyst. Data were analyzed using deductive thematic analysis [25]. Open coding was used, and codes were developed and modified as the analysis took place. Analyzing the codes enabled the identification of initial themes; these preliminary themes were refined to demonstrate interesting patterns in the data that were important to the successes or failures of the implementation. Themes were realized semantically (i.e. the explicit or surface meaning of the data) and latently, to identify and examine underlying ideas and assumptions that inform the semantic content of the data [26].

### Bland-Altman plots

Bland-Altman plots were created in GraphPad Prism 8 on the log-transformed copy number per 5μL data. Log transformation was performed to normalize data (Shapiro-Wilk test, test statistic 0.22, p < 0.00). [27,28].

### Statistics

Statistical analyses were conducted in IBM SPSS (version 25, USA). Graphs were made in Prism 8 (GraphPad, USA). Limit of Detections were calculated using the POD/LOD calculator [29]. Maximum log difference was calculated as the upper 95% confidence interval of the average of the log difference between all sets of paired samples. Interclass correlation analysis was performed in SPSS on the log-transformed data using a two-way random-effects model with average measures, and a type c model with a consistency definition. A two-way random-effects model was selected because it models both an effect of the operator and the sample and assumes that both are drawn randomly from larger populations.

## Results

### Thermocycler comparison

#### Detection limits of the open qPCR thermocyclers

The limit of detection 95 (LOD_95_) of the Open qPCR thermocyclers is 63.4 gene copies (GC)/5μL (lower limit 43.7 GC/5μL, upper limit 89.2 GC/5μL, n = 40, based on all qPCR tests). This is approximately 1-log higher than the same assays (Avian schistosomes LOD_95:_ 3.4 GC/5μL; Toxic cyanobacteria LOD_95_: 6.25 GC/5μL; HF183 LOD_95_: 7.2 GC/5μL) performed using our laboratory ABI 7500/QuantStudio 3 thermocycler. All of these assays have been validated in previous papers, the names, sequences, and the references for the primers and probes are found in S1 Table. Standard curves performed well compared to the optimal qPCR standard curve (i.e. slope: -3.3, amplification factor: 1.9–2.0, R^2^: 0.99) using the Open qPCR thermocyclers [30] (S1 Table).

#### Comparison between machines

Interclass correlation coefficients (ICC) were calculated to compare CBM partner DNA extracts run on the Chaibio Open qPCR machine, and our laboratory ABI 7500/QuantStudio 3. In 2017, the ICC of the avian schistosomes assay was 0.88 (95% CI: 0.85 lower, 0.90 upper), and in 2018, it was 0.76 (95% CI: 0.56 lower, 0.866 upper), in 2018 this group used 2 Open qPCR machines, and this ICC is a pooled result of both of these machines. In 2018, the ICC of the toxic cyanobacteria assay was 0.57 (95% CI: 0.1 lower, 0.86 upper) (Table 1). Maximum log differences were also calculated and ranged from 1–1.5 depending on the test and year (Table 1). CBM partners also prepared sample blanks and performed DNA extraction and qPCR on these. All sample blanks were negative when performed both in the field and on the more sensitive core laboratory equipment.

**Table 1:** Interclass correlation coefficients and maximum log difference. Comparing the reproducibility of samples run on the Chaibio Open qPCR thermocycler and the ABI 7500 thermocycler/QuantStudio 3.

	**Comparison of Partner-Extracted DNA Samples Performed On The Open qPCR Versus The QuantStudio 3/ABI 7500**				
**qPCR Test**	**Interclass correlation coefficient**	**Lower 95% CI**	**Upper 95% CI**	**Maximum log difference**	**N**
Toxic cyanobacteria 2018	0.57	0.1	0.86	1.2	12
Toxic cyanobacteria 2019	0.6	0.24	0.8	1.5	40
Avian schistosomes 2017	0.88	0.85	0.9	1	255
Avian schistosomes 2018	0.76	0.56	0.87	1	47
	**Comparison of Partner-Extracted And Expert-Extracted Split Samples**				
	**Interclass correlation coefficient**	**Lower 95% CI**	**Upper 95% CI**	**Maximum log difference**	**N**
Toxic cyanobacteria 2018	0.65	-0.25	0.9	1.4	12
Toxic cyanobacteria 2019	0.67	0.366	0.83	1.3	39
Avian schistosomes 2017	0.54	0.32	0.68	1.4	255
Avian schistosomes 2018	0.59	0.34	0.75	1.3	70

### CBM partner comparison

#### Semi-quantitative analysis using Bland-Altman plots

**Fig 2:**
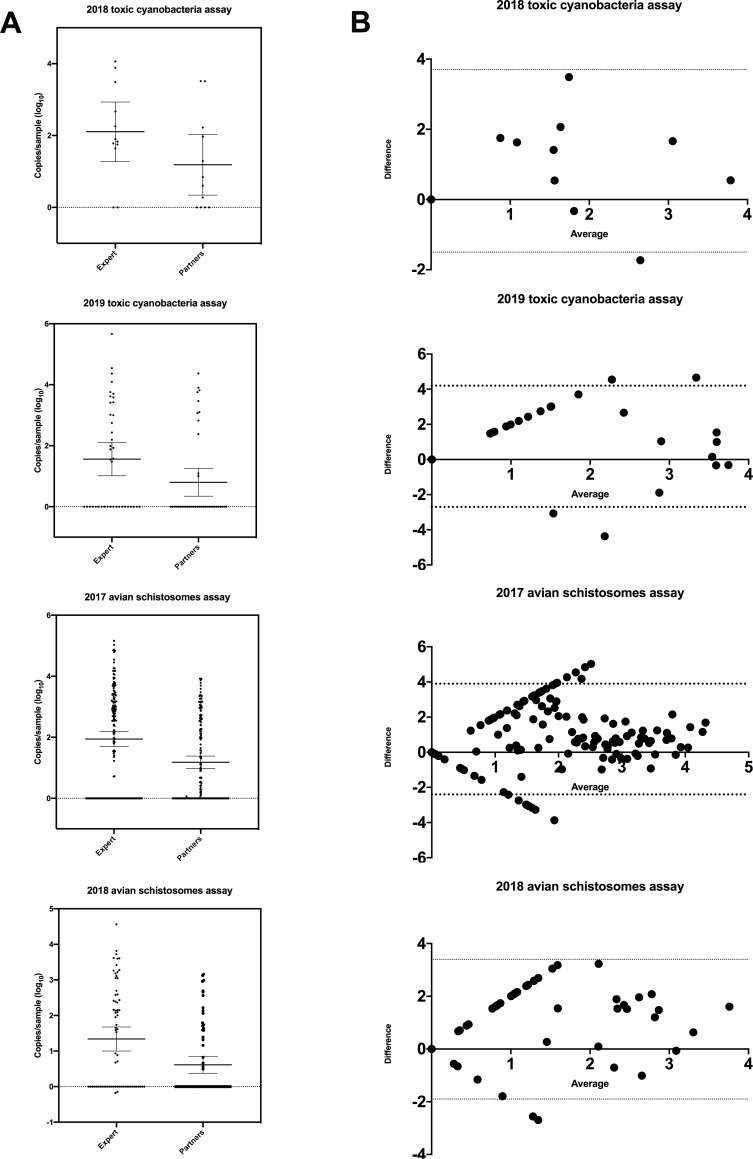
Bland-Altman graphs of the difference between the CBM partners' data and the central laboratories’ data. Limits of agreement (1.96 times the standard deviation) are bounded by the dotted lines. Top: Agreement of the 2017 Avian schistosome monitoring program. Middle: Agreement of the 2018 avian schistosomes monitoring program. Bottom: Agreement of the microcystin gene monitoring program.

Reproducibility was assessed using the semi-quantitative Bland-Altman plot. Bland-Altman plots graph the average of two measurements on the X-axis and the difference between these measurements on the Y-axis. The Bland-Altman plot for avian schistosomes monitoring for 2017 and 2018 show a linear pattern at lower copy numbers, but at higher copy numbers show uniform variability (Fig 2). Bland-Altman analysis of the toxic cyanobacteria test shows uniform variability within the limits of agreement (1.96 times the standard deviation). A paired t-test using the log-transformed data was used to compare the within-subject standard deviations of the partner data compared to the laboratory-generated data. They were significantly different based on an F-test and Welch’s t-test (p < 0.0001, F = 6288, mean difference ± SEM: 20326 ± 9843).

#### Interclass correlation analysis

ICC analysis was performed to compare user and laboratory samples. In 2017, the Biomeme MI extraction kit was used for swimmer’s itch monitoring. The ICC between user and laboratory extraction samples was 0.539 (95% CI: 0.320 lower, 0.680 upper). The ICC 2018 for avian schistosomes monitoring was 0.593 (95% CI: 0.344 lower, 0.747 upper). The ICC *mcyE* was 0.640 (95% CI: -0.250 lower, 0.896 upper) (Table 1). Maximum log differences ranged from 1.3–1.4 (Table 1).

#### Inhibition controls

PCR Inhibition was tested on partners' DNA extractions and on DNA extractions performed in house. Five percent (49 samples) of samples were inhibited (as defined by a 3-ct shift in the inhibition control) in both partner and in house extractions. Inhibited samples were excluded from the analyses in this paper because inhibitors will alter the estimates of copy number when present.

### Qualitative analysis

#### User perceptions

User perceptions of the program were captured through a written survey that was administered to participants. The questions are available in S4 Table. Thirty-three percent (33%) of respondents stated that they had some prior knowledge of molecular biology, PCR (polymerase chain reaction), eDNA, or DNA based detection in general prior to the use of the qPCR field method. Fifty percent (50%) reported having low prior knowledge, and one participant had no prior knowledge. The same 33% of respondents who reported some knowledge with molecular biology and methods also reported having performed some form of PCR in the past. The rest of the respondents reported not having performed PCR (50%), and one participant did not remember. However, prior knowledge did not impact the training all users were provided.

#### Thematic analysis

User surveys underwent deductive thematic analysis whereby surveys were coded, and then codes were organized into themes [26]. The codes identified and relevant excerpts from the surveys are presented in S5 Table. The first theme identified is “rapidly responding to hazards.” This theme captured the CBM partners’ perceptions of the speed of the qPCR method and their perceived ability to respond to issues quickly. The second theme identified was the question of who controls the CBM monitoring system. This theme emerged from CBM partners expressing a desire for independence and control over the interpretation of results. The third theme identified was that the triangulation of training was valuable in that most CBM partners suggested that the written and video protocols (complemented with a few in-person training sessions) were important to them and enhanced their learning. A subtheme that emerged from this theme was “learning and communication.”

## Discussion

### Implementation of the CBM qPCR program

We assessed the accuracy of the portable qPCR machines relative to a “core” machine, and the ability for CBM partners to execute the method. Our analyses have demonstrated that a CBM qPCR monitoring program can yield accurate results for different targets (i.e., eukaryotic versus prokaryotic) when deployment of the method is controlled.

Our intention was to implement a CBM qPCR system in a real-world context. As Fig 1 details, we began the development of this project by consulting with local stakeholder groups and assessing their interest in the project and what types of biological hazards and surrogates they might be interested in monitoring for. Our goal was to have partners run a sufficient number of tests so as to test the reproducibility of the method. We did not want to prescribe a particular test for CBM partners to run, as if a particular test was not useful to a partner, it would be unlikely that they would be motivated to continue testing. Therefore, we adapted to the needs of our CBM partners and adapted a variety of existing qPCR tests to the field equipment and testing protocol. Additionally, some of the groups we worked with had their own scientific questions they wanted to answer, and we facilitated this.

Our laboratory distributed all materials required to complete testing to users. Additionally, we prepared all qPCR master mix components (enzyme mix, primers and probes) and aliquoted these into individual reaction tubes for users. The purpose of this was two-fold, to prevent contamination of CBM partners’ qPCR reactions and for simplicity for partners. Our laboratory facilities are equipped with a PCR clean room, as well as separate pre and post-amplification rooms. By preparing reaction tubes and controls, we could prevent CBM partners from handling high copy number controls (a likely source of contamination). Additionally, CBM partners were instructed not to open tubes that had undergone qPCR. The Biomeme DNA extraction does not utilize pipettes, but all users were supplied with filter-tipped 20μL pipettes to add their purified DNA into their reaction tubes. Pre-preparing reaction tubes made running qPCR as simple as adding the DNA and pressing “Start” on the Open qPCR machine. Our laboratory also performed an analysis of the qPCR data. CBM partners would download their spreadsheets from the Open qPCR and send them either via email or Google Drive to our laboratory, where we would analyze control data, and calculate copy numbers and, where possible, organismal numbers for partners. Again, this was done in an attempt to preserve the simplicity of the method, and because analysis of qPCR data is complex and requires an expert eye.

The CBM partners participating in this study ran 985 total samples over the two years of this program. Deductive thematic analysis was performed to analyze CBM partner surveys, which is a method of analysis by which codes and theme development were directed by our existing research questions. Three primary themes emerged from this analysis.

The first theme identified was “Rapidly responding to hazards.” Our CBM partners liked that the “time requirement from the qPCR testing method was less than the traditional operational time frame…" Our CBM partners seemed to equate the rapidness of the method to a rapid policy response to hazards. This was not the case, as our province has only begun to adapt policy frameworks to qPCR methods.

The second theme was “Independence and verification of a CBM monitoring system.” CBM partners expressed a desire for more independence and more control over the interpretation of results. Our study was designed to remove data interpretation from the participant’s hands. We thought this would be beneficial because the interpretation of qPCR data is not trivial, and to prevent panic if CBM partners saw positive samples that, while meaningful, might not constitute a real concern. CBM partners said they wished the data was published online, "If the data was available or if there was a way to input the data online into a database. Then we could use the results more easily,” they said. Our CBM partners also expressed a desire to validate their results and have access to quality control data. One user suggested, "…a visual that compared our results to yours, so we have some idea of if we were capturing the results accurately." Another specifically suggested that "…third party verification can be one method to enhance the validity of the results," suggesting a desire for some oversight to ensure data quality, but also a desire for CBM partners to know that they are contributing meaningful and accurate results.

One of the biggest challenges for CBM programs is data validation, storage, and visualization. However, tools are emerging to address this challenge, including the Lake Observer mobile app through the Global Lake Ecological Observatory Network, the DataStream through the Gordon Foundation, or the ABMI’s NatureLynx. Allowing community partners to upload and visualize their results may help partners feel that they are part of something bigger. It may allow them to contextualize their results relative to other water bodies, log additional environmental observations, or upload photographs of recreational waters. These apps can also be helpful to track long-term results or to have the data incorporated into reporting by other agencies.

The third theme we identified was that the triangulation of training was valuable. CBM partners appreciated the three forms of training. Most CBM partners found "the training videos were really useful." CBM partners found the written protocol useful as a reference but suggested that after "around 2–3 runs of the machine, this resource was no longer needed." Most CBM partners stressed the importance of the in-person training, and one user stated that "the in-person training went a long way in creating and (sic) increased comfort and confidence in the machine." Other studies that have looked at how to effectively train participants for CBM projects have found that multiple training sessions can improve data accuracy [31]. A recent study that focussed specifically on training in molecular biology especially emphasized that multiple training sessions and especially hands-on training was key to participants being able to successfully complete the method [32].

### Agreement between CBM partners and scientists

The LOD_95_ is the lowest concentration of DNA that can be reliably detected in 95% of samples; it is a measure of sensitivity. The Open qPCR thermocycler has a higher limit of detection when using a Taqman fluorescein probe than the ABI core thermocyclers (63.4 DNA copies/5μL versus >10 DNA copies/5μL across all methods, respectively). The field thermocyclers are less sensitive than the core laboratory machine. Understanding this change in the detection limit is important in determining if the CBM qPCR system would be effective for a particular test. For instance, if the concentration of the target that might constitute a risk is below the LOD_95_ for the Open qPCR thermocyclers, “risky” samples will appear negative as this thermocycler is not capable of detecting them. When we deployed the human-associated Bacteroides HF183 CBM testing for recreational shoreline source tracking in Michigan, USA, our CBM partners reported only a single positive sample. However, when these DNA extracts were analyzed, 22.7% (54/237), were found to be positive for between 15–35 copies DNA/5ul. Seven of these samples approached the LOD_95_ of the Open qPCR thermocycler, and CBM partners detected one of these samples. This is a common issue in PCR based fecal source tracking studies, not just in CBM studies, and highlights the importance of ensuring a particular method fits the research question, especially in a CBM study [33]. Nonetheless, a recent study found that an HF183 gene copy number of 4200 HF183/100ml exceeds the USEPA benchmark risk of GI illness [10]. This level is equivalent to a gene copy number of 210 HF183 GC/5ul—well above the detection limit of the Open qPCR thermocycler. Thus, outbreak scenarios would be clearly discernable. However, this also illustrates an example of how the monitoring project must be clearly rooted in a management outcome. If the intention of the monitoring program is to detect potential outbreak scenarios and initiate action, the increased detection limit is acceptable. However, if the management context is the detection of leaking septic areas, or source tracking fecal markers on a beach, this detection limit would be inappropriate. It is important to work closely with CBM partners to understand their specific monitoring questions, and critically appraise if CBM qPCR is capable and appropriate to answer these questions.

ICC analysis for the avian trematode assays showed a very high level of agreement between the Open qPCR thermocycler and the core thermocyclers (Table 1). The toxic cyanobacteria test showed much lower levels of agreement between the field thermocycler and the laboratory thermocycler. We discovered through analyzing the control standards that the heated lid on the field thermocycler was loose and therefore was failing to engage properly with the tops of the reaction tubes (i.e. machine failure). However, from a quality control perspective, we were able to detect a probable machine failure with a sample size comparison of 11. This is extremely promising for future larger-scale CBM qPCR systems. It suggests that it would be possible with a relatively low number of samples being confirmed by a core facility or quality control partner to detect user or machine error once a baseline level of agreement for a single test had been established.

The comparison between CBM partners performing DNA extraction and experts performing the DNA extraction was first assessed semi-quantitatively using the Bland-Altman plot (Fig 2). The results of this analysis for the almost all targets show a linear and negative linear pattern at lower gene copy numbers. This can be due to bias between methods, but can also be caused by a difference in the within-subject standard deviation [28]. This seems plausible as users with potentially very different skill levels are performing the two methods. A paired t-test using the log-transformed data was used to compare the within-subject standard deviations. They were significantly different, which suggests that the linear pattern observed is due to increased variability in CBM partner data.

Partner extracted samples are typically lower in copy numbers than expert-extracted samples (Fig 2). This is likely due to differences in DNA extraction efficiency between the CBM partners and experts. However, it seems more experienced users become better at DNA extraction over time, as both the avian schistosomes monitoring group and the toxic cyanobacteria monitoring groups seem to improve over time. (Fig 2). Its unsurprised that the ICCs and maximum log differences are higher when comparing partner and expert extracted DNA samples due to the highly variable nature of DNA extraction, and because the duplicate samples run in the central laboratory could never be expected to contain exactly the same amount of organism. The ICCs of the DNA extraction comparison ranged from 0.54 to 0.67, with maximum log differences ranging from 1.3 to 1.4 (Table 1). It is important to note that for the avian schistosomes monitoring program, a change was made in 2018 to establish a fully functional remote laboratory, and move these partners onto using the Qiagen DNeasy DNA extraction kit. This change was made at the request of the CBM partners, who would typically collect and analyze hundreds of samples each field season. Details about the equipment in this satellite laboratory can be found in S2 Table.

Ebentier et al. (2013) conducted a reproducibility analysis of five core laboratories on a panel of microbial source tracking qPCR markers. They calculated reproducibility as the maximum expected log difference (within 95% confidence) between the different laboratories. Their analysis demonstrated reproducibility coefficients for different qPCR assays were highly variable, between 0.09–0.66 log. The methods that were likely to produce higher copy numbers, like Enterococcus qPCR testing via USEPA Method 1611, showed higher reproducibility coefficients than methods that were likely to produce lower copy numbers, like human-associated Bacteroides marker HF183. They also analyzed the contribution to variability of a variety of factors (the sample itself, equipment, procedures) to the measurement. Their paper concluded that when protocols and reagents were not standardized, the agreement between methods decreased. They highlighted the need for standardization of protocols and consumables before the implementation of studies involving multi-laboratory experiments [34,35].

The maximum log difference of the CBM qPCR monitoring program higher than the values reported in the Ebentier paper. Reproducibility between the same extract performed by our expert team and the CBM partners ranged from 0.44 to 1.5 log, and reproducibility coefficients of between partner and expert extracted split samples ranged from 1.3 to 1.4 log (Table 1). It should be noted that the majority of the qPCR methods deployed routinely detected copy numbers in excess of 1 log. Thus, we might expect higher variability between replicates at these larger copy numbers (Fig 2). CBM qPCR monitoring programs will likely generate data that does have higher variability. It’s important to weigh the pros of a CBM qPCR approach, notably that a CBM qPCR approach may result in increased numbers of samples from across a larger geographic area and builds relationships and partnerships across sectors.

### Future directions for the CBM qPCR program

Rapid monitoring approaches, including CBM qPCR, should be deployed within the context of a policy framework and management response plan that can support acting upon the results generated. The response plan for samples that might constitute a hazard should be clear to CBM partners. If response plans lack transparency, a CBM partner who encounters a sample that contains a high level of an indicator organism, but upon subsequent tests shows low or no risk, might be dismayed by a lack of response by government. A CBM qPCR monitoring system in recreational water would need to prioritize communication and understanding between regulators and CBM partners, and would likely function best when addressing specific objectives [36].

Whether the rapid CBM qPCR monitoring system enables a more rapid response to hazards is yet to be seen; however, CBM qPCR monitoring certainly has the advantage of being able to generate data over a large geographic area and for numerous hazards. It could be adapted to measure organisms not typically considered in monitoring programs; as we have demonstrated in our study, the approach works equally well for eukaryotic hazards like parasitic organisms as it does for the more traditional prokaryotic targets like enteric bacteria. The flexibility inherent in CBM qPCR makes this an attractive and adaptive platform for governments and communities to answer management related questions for their watersheds.

Our vision for the CBM qPCR monitoring system was that data analysis would not occur in the hands of CBM partners (Fig 1). Analysis of qPCR data, while not extremely complex, does require a more comprehensive understanding of qPCR data; additionally, data interpretation is typically the most erroneous component of CBM programs [37,38]. Our study supports a CBM monitoring program that is supported by a central agency. Some central program (i.e., academic, governmental, or not-for-profit) should distribute materials and provide QC support. Participants in our study expressed a desire to know how well they were performing the method. This highlights an important component of a large-scale CBM monitoring program: a compliance testing system that would test and train potential participants to ensure the method is being conducted appropriately. This must include third-party verification of a certain percentage of all samples tested. While verification is important to ensure CBM partners are generating reliable results, it is essential that communication is prioritized. This includes responding quickly to results reported by CBM partners when a potential hazard is detected. It also includes being honest with partners about their performance, and willingness by both the CBM and regulatory partners to collect and assess additional samples when clarification or confirmation is required. Any CBM program should support and empower communities to answer monitoring and research questions they are interested in.

## Conclusion

To our knowledge, this is the first study to comprehensively test the accuracy of a CBM qPCR water monitoring approach in a real-world context. Our results show that when implemented in a controlled manner, such that a central body controls materials and protocols, results can be highly reproducible. Our study also suggests that CBM partners, whose buy-in would be required for ensuring program longevity, value the method and the results it provides.

CBM qPCR could process a large number of samples from a wide geographical area that could aid beach management for health and invasive species. CBM qPCR could act as a valuable component of an environmental monitoring surveillance system. qPCR is a platform, and therefore a myriad of diagnostic tests could be deployed as needed in remote locations. While CBM qPCR programs may be more variable than traditional monitoring programs, they could serve as a comprehensive screening system for traditional monitoring programs. In many contexts, CBM qPCR programs could be as accurate as traditional testing and have the potential to replace traditional testing.

## Supporting information

S1 Table Primers and probes used in this study. (DOCX)

S2 Table Standard curves of each assay performed on the open qPCR and the core laboratory machine. Data shown represent the average of 5 runs (each consisting of two internal replicates of each standard). Ideal standard curves have an efficiency of between 0.98 and 0.99, a slope of -3.32, and an efficiency of 100%, which can also be represented as an amplification factor of 2, which suggests product has doubled every cycle.
(DOCX)

S3 Table Field method materials used in this study. (DOCX)

S4 Table Questions administered to users in survey. (DOCX)

S5 Table (DOCX)
