# Supplementary material for: Democratizing water monitoring: Implementation of a community-based qPCR monitoring program for recreational water hazards
Source: PLoS One. 2020 May 13;15(5):e0229701. doi: 10.1371/journal.pone.0229701 (PMC7219769; doi:10.1371/journal.pone.0229701)
Supplement: S1 Table — (DOCX) [file pone.0229701.s001.docx]

| **Name** | **Sequence (5'-3')** | **Reference** |
| --- | --- | --- |
| **Toxic cyanobacteria (mcyE gene targeting)** | | |
| 127 Fwd | AAGCAAACTGCTCCCGGTATC | Qiu et al., 2015 |
| 186 Probe | /FAM/CAATGGTTAT/ZEN/CGAATTGACCCCGGAGAAAT /IABkFQ | Qiu et al., 2015 |
| 247 Rev | CAATGGGAGCATAACGAGTCAA | Qiu et al., 2015 |
| **Avian trematode (18S gene targeting)** | | |
| JVSF 18S Fwd | AGCCTTTCAGCCGTATCTGT | Narayanan et al., 2015 |
| JVSP 18S Probe | /FAM/AGGCC/ZEN/TGCCTTGAGCACT/IABkFQ/ | Narayanan et al., 2015 |
| JVSR 18S Rev | TCGGGAGCGGACGGCATCTTTA | Narayanan et al., 2015 |
| **Human associated bacteroides HF183 (16S targeting)** | | |
| HF183 Fwd | ATCATGAGTTCACATGTCCG | Haugland et al., 2010. |
| BFDProbe | FAM/CTGAG/ZEN/AGGAAGGTCCCCCACATTGGA/IABkFQ/ | Haugland et al., 2010. |
| BFDRev | CGTAGGAGTTTGGACCGTGT | Haugland et al., 2010. |

**S1 Table: Primers and Probes used in this study.**
