# Supplementary material for: Democratizing water monitoring: Implementation of a community-based qPCR monitoring program for recreational water hazards
Source: PLoS One. 2020 May 13;15(5):e0229701. doi: 10.1371/journal.pone.0229701 (PMC7219769; doi:10.1371/journal.pone.0229701)
Supplement: S2 Table — Data shown represent the average of 5 runs (each consisting of two internal replicates of each standard). Ideal standard curves have an efficiency of between 0.98 and 0.99, a slope of -3.32, and an efficiency of 100%, which can also be represented as an amplification factor of 2, which suggests product has doubled every cycle. (DOCX) [file pone.0229701.s002.docx]

**S2 Table. Standard curves of each assay performed on the Open qPCR and the core laboratory machine.** Data shown represent the average of 5 runs (each consisting of two internal replicates of each standard). Ideal standard curves have an efficiency of between 0.98 and 0.99, a slope of -3.32, and an efficiency of 100%, which can also be represented as an amplification factor of 2, which suggests product has doubled every cycle.

|  | Copy number in standard | Cycle threshold (average, st dev) | | r2 | Slope | Amplification  factor |
| --- | --- | --- | --- | --- | --- | --- |
|  |  |  |  |  |  |  |
| Cyanobacteria mcyE assay | | | | | | |
| Open qPCR | 5000 | 30.1 | 0.27 | 0.980 | -3.05 | 2.100 |
|  | 500 | 33.2 | 1 |  | | |
|  | 50 | 36.3 | 1.6 |  |  |  |
| ABI qPCR | 5000 | 28.7 | 1.5 | 0.990 | -3.700 | 1.800 |
|  | 500 | 32.2 | 2.4 |  | | |
|  | 50 | 36.1 | 0.91 |  |  |  |
| Human-associated bacteroides (HF183) assay | | | | | | |
| Open qPCR | 5000 | 25.6 | 0.25 | 0.99 | -3.46 | 1.94 |
|  | 500 | 29.2 | 0.24 |  | | |
|  | 50 | 32.9 | 0.28 |  |  |  |
| ABI qPCR | 5000 | 24.8 | 0.13 | 0.98 | -4.4 | 1.87 |
|  | 500 | 28.1 | 0.31 |  | | |
|  | 50 | 33.7 | 0.26 |  |  |  |
| Pan-avian schistosome assay | | | | | | |
| Open qPCR | 5000 | 27.3 | 0.41 | 0.99 | -3.03 | 2.1 |
|  | 500 | 30.3 | 0.64 |  | | |
|  | 50 | 33.3 | 0.52 |  |  |  |
| ABI qPCR | 5000 | 26.9 | 0.52 | 0.99 | -3.03 | 2.1 |
|  | 500 | 30.5 | 0.47 |  | | |
|  | 50 | 32.9 | 0.54 |  |  |  |
