# Supplementary material for: Democratizing water monitoring: Implementation of a community-based qPCR monitoring program for recreational water hazards
Source: PLoS One. 2020 May 13;15(5):e0229701. doi: 10.1371/journal.pone.0229701 (PMC7219769; doi:10.1371/journal.pone.0229701)
Supplement: S3 Table — (DOCX) [file pone.0229701.s003.docx]

**S3 Table**. Field method materials used in this study

| **Name** | **Manufacturer/Catalogue Number** |
| --- | --- |
| Biomeme MI DNA extraction kit | Biomeme |
| DNAeasy DNA extraction kit | Qiagen (69506) |
| 0.4 uM filters | Pall (FMFNL1050) |
| Vaccum pump | Vaccubrand ME 1C |
| PCR tubes and caps | Axygen (PCR-02-FCP-C and PCR-0108-LP-C) |
| Microcentrifuge tubes | Eppendorf (Z666548-250EA) |
| Open qPCR thermocycler (single channel) | ChaiBio. |
| 20 μL Micropipette | VWR (470231-608) |
| Maximum Recovery Filter tips | Axygen (TF-20-L-R-S) |
| Pelican Storm Case | Pelican IM2450 |
| 20uM Plankton Tow | Acquatic Research Instruments |
| Primetime Gene Expression MasterMix | IDTDNA (1055772) |
| Computers | Google Chromebook and Acer Switch One. |
| **Field Laboratory Equipment used to perform Qiagen DNAeasy DNA Extraction** | |
| Spectrafuge 24D | Dot scientific (C2400) |
| VorTemp 56 Shaking Incubator | Dot scientific (S2056) |
| Mortexer™ | Dot scientific (BV-1005) |
| Mortexer™ 12 sample head | Dot scientific (BV1000-H15) |
