# Supplementary material for: Democratizing water monitoring: Implementation of a community-based qPCR monitoring program for recreational water hazards
Source: PLoS One. 2020 May 13;15(5):e0229701. doi: 10.1371/journal.pone.0229701 (PMC7219769; doi:10.1371/journal.pone.0229701)
Supplement: S4 Table — (DOCX) [file pone.0229701.s004.docx]

**S4 Table. Questions administered to users in survey**

| **Section 1. Prior Experience** | |
| --- | --- |
| 1. How would you rank your knowledge with molecular biology,PCR (polymerase chain reaction),eDNA or DNA based detection in general  prior to the use of the qPCR field method | |
| o | High prior knowledge |
| o | Some prior knowledge |
| o | Low prior knowledge |
| o | No prior knowledge |
| 2. Had you performed PCR (polymerase chain reaction) before attempting the qPCR field method? (highlight your response) | |
| o | Yes |
| o | No |
| o | I do not remember |
| **Section 2. Training** | |
| 3. Was the training on the qPCR field-testing sufficient? | |
| 3a. Did you utilize the written protocol/video? | |
| 3b. Was the in-person training valuable? | |
| **Section 3. DNA extraction** | |
| 4a. Was the DNA extraction protocol simple? | |
| 4b.What could be improved? | |
| **Section 4. Operating the thermocycler** | |
| 5. Did you find the operation of the Chai Bio Open qPCR (the thermocycler and computer) simple?  What could be improved? | |
| 6. What method of results reporting would you have liked to see? | |
| **Section 5.** | |
| 7. Was the time spent on the method too long or just right? | |
| 8. Did the portable field qPCR unit fit well within your/your organizations normal monitoring tasks? | |
| 9. Did the portable field system meet your expectations? | |
| 10. Did you find the results obtained by qPCR valuable? What could make these results more valuable? | |
| 11. Do you think that field qPCR is appropriate to answer the questions your organization  sought to answer in agreeing to participate in this trial? | |
| 12. Do you see any value for continued use of the field qPCR method within your organization? | |
| 13. Why were you interested in participating in this trial to begin with? Have your views on the use  of field qPCR changed since then? | |
