# Supplementary material for: Democratizing water monitoring: Implementation of a community-based qPCR monitoring program for recreational water hazards
Source: PLoS One. 2020 May 13;15(5):e0229701. doi: 10.1371/journal.pone.0229701 (PMC7219769; doi:10.1371/journal.pone.0229701)
Supplement: S5 Table — (DOCX) [file pone.0229701.s005.docx]

| **Theme** | Rapidly responding to hazards | | Triangulation of training | | | | Independence and verification of the CBM monitoring system | | | | |
| --- | --- | --- | --- | --- | --- | --- | --- | --- | --- | --- | --- |
| **Subtheme** | | |  | | | | Learning new methodologies and technologies | | | | |
| **Code** | Rapid response | Rapid method | In-person training | Troubleshooting | Written protocol | Videos | Quality control | Independence | Learning | Communication | Value |
| **Examples** | "..implemented as an invasive species monitoring plans (sic) that poses to be 'faster' than traditional methods" | "yes the time requirement [spent on the method] was good" | "the in-person training went a long way in creating and(sic) increased comfort and confidence in the machine" | "we had some software issues but they were resolved." | "around 2-3 runs of the machine this resource was no longer needed" | "yes, the training videos were really useful!" | "…a visual that compared our results to yours so we have some idea of if we were capturing the results accurately" | "…more detail is always nice to see in the results in the sense that you might be able to explain what is going on or provide a reasonable account at least" | "I am always up for learning new methodologies to answering scientific questions" | "…if there was an online form we could fill in as we go so there is no confusion about what we were doing.." | "used in the right way and right circumstance can be extremely valuable." |
|  | "..able to give us quick updates on lakes that were of concern or maybe did not have [health monitoring] samples being taken frequently." | "…time requirement from the qPCR testing method was less than the traditional operational time frame.." | "Talking in person about the procedure where I could ask real time questions was definitely the most helpful in learning the procedure" | "a troubleshooting component with the machine if they have any issues" | "They were a helpful reference tool" | "at the end of those videos I still had quite a few questions about the techniques" | "non-specialized individuals could get accurate results In a short time frame" | "If the data was available or if there was a way to input the data online into a database. Then we could use the results more easily" | "always interested in integrating new technologies into monitoring protocols" | "helpful for people who recreate on a lake that isn’t a formal recreation site included in the [health monitoring program] sampling" | "It is much more sustainable and cost effective than [traditional methods]." |
|  | "It was awesome to get real time data that we could use the next day to change our field procedures and experimental designs" | "just right. The filtering is what took the longest" | "I believe the this training allowed for an appropriate extent od certainty when visually examining the results output by the qPCR machine" | "I didn't have a lot of 'problem solving' skills myself with the program, if something was going wrong, I didn’t know what to do/how to analyze the data without calling for help. It would have felt more empowering… if I had some sort of a 'troubleshooting' type document.. I might have been able to solve some of the problems on my own." |  |  | "I believe that third party verification can be one method to enhance validity of the results" | "...we made a shortened direction sheet ourselves--other people might also find that helpful if they don’t have it? We mad a bulleted type list with just keywords/amounts." | "I’ve come to appreciate qPCR more and more, the more I learn" | "quick results to communicate to our volunteers" | "it was the perfect tool and was instrumental in all the success we are having, couldn't be happier" |
|  | "The testing was essential for quick responsive results to ensure public safety" | "results were valuable and quicker than lab samples" | "..helpful to received training reading material before the in-person training to prime the actual training." |  |  |  |  |  |  |  |  |
|  | "allowed us to be responsive and more proactive to ensure public safety" |  |  |  |  |  |  |  |  |  |  |
